# Orbital stability analysis and photometric characterization of the second Earth Trojan asteroid 2020 XL_5_

**DOI:** 10.1038/s41467-022-27988-4

**Published:** 2022-02-01

**Authors:** T. Santana-Ros, M. Micheli, L. Faggioli, R. Cennamo, M. Devogèle, A. Alvarez-Candal, D. Oszkiewicz, O. Ramírez, P.-Y. Liu, P. G. Benavidez, A. Campo Bagatin, E. J. Christensen, R. J. Wainscoat, R. Weryk, L. Fraga, C. Briceño, L. Conversi

**Affiliations:** 1 Departamento de Fisica, Ingeniería de Sistemas y Teoría de la Señal, Universidad de Alicante, Carr. de San Vicente del Raspeig, s/n, 03690 San Vicente del Raspeig, Alicante, Spain; 2 Institut de Ciències del Cosmos (ICCUB), Universitat de Barcelona (IEEC-UB), Carrer de Martí i Franquès, 1, 08028 Barcelona, Spain; 3 ESA NEO Coordination Centre, Largo Galileo Galilei, 1, 00044 Frascati, Italy; 4 Arecibo Observatory, University of Central Florida, HC3 Box 53995, Arecibo, PR 00612 USA; 5 Instituto de Astrofísica de Andalucía, CSIC, Apartado 3004, 18080 Granada, Spain; 6 Instituto de Física Aplicada a las Ciencias y las Tecnologías, Universidad de Alicante, San Vicente del Raspeig, 03080 Alicante, Spain; 7 Observatório Nacional / MCTIC, R. Gen. José Cristino, 77, Rio de Janeiro, 20921-400 Brazil; 8 Astronomical Observatory Institute, Faculty of Physics, A. Mickiewicz University, Słoneczna 36, 60-286 Poznań, Poland; 9 Solenix Deutschland GmbH, Spreestraße 3, 64295 Darmstadt, Germany; 10 The University of Arizona, Lunar and Planetary Laboratory, 1629 E University Blvd, Tucson, AZ 85721 USA; 11 Institute for Astronomy, University of Hawaii, 2680 Woodlawn Dr, Honolulu, HI 96822 USA; 12 Department of Physics and Astronomy, The University of Western Ontario, 1151 Richmond St, London, ON N6A 3K7 Canada; 13 Laboratório Nacional de Astrofísica LNA/MCTIC, R. dos Estados Unidos, 154, Itajubá, 37504-364 Brazil; 14 Cerro Tololo Inter-American Observatory/NSF’s NOIRLab, Casilla 603, La Serena, Chile; 15 ESA ESRIN, Largo Galileo Galilei, 1, 00044 Frascati, Italy

**Keywords:** Asteroids, comets and Kuiper belt, Inner planets

## Abstract

Trojan asteroids are small bodies orbiting around the *L*_4_ or *L*_5_ Lagrangian points of a Sun-planet system. Due to their peculiar orbits, they provide key constraints to the Solar System evolution models. Despite numerous dedicated observational efforts in the last decade, asteroid 2010 TK_7_ has been the only known Earth Trojan thus far. Here we confirm that the recently discovered 2020 XL_5_ is the second transient Earth Trojan known. To study its orbit, we used archival data from 2012 to 2019 and observed the object in 2021 from three ground-based observatories. Our study of its orbital stability shows that 2020 XL_5_ will remain in *L*_4_ for at least 4 000 years. With a photometric analysis we estimate its absolute magnitude to be ${H}_{r}=18.5{8}_{-0.15}^{+0.16}$, and color indices suggestive of a C-complex taxonomy. Assuming an albedo of 0.06 ± 0.03, we obtain a diameter of 1.18 ± 0.08 km, larger than the first known Earth Trojan asteroid.

## Abstract (web summary)

Although Trojan asteroids have been known for decades in other Solar System planets, only one Earth Trojan asteroid was detected. Here, the authors show that recently discovered 2020 XL_5_ is the second transient Earth Trojan asteroid.

## Introduction

The classical work of J. L. Lagrange published in 1772 on the three-body problem had to wait until 1906 to find an empirical verification with the discovery of asteroid (588) Achilles. This asteroid is orbiting around a theoretical point located 60° ahead of Jupiter along its orbit. After discovering (588) Achilles, many other objects were found orbiting around nearly the same point or its mirroring position 60° behind Jupiter. Both points are the so-called triangular Lagrange points and are commonly known as *L*_4_ (for the former) and *L*_5_ (for the latter)^1^. Asteroids orbiting around these points of a planet-Sun system are known as Trojan asteroids.

Although Trojan asteroids have been known for decades in other Solar System planets^2^ such as Venus^3^, Mars^4^, Jupiter^5^, Uranus^6^, and Neptune^7^, it wasn’t until 2011 that asteroid 2010 TK_7_ was found to be the first (and hitherto unique) Earth Trojan (ET) asteroid^8^. ET asteroids have been broadly debated and proved to be feasible from the point of view of celestial mechanics. In particular, their possible existence has been shown using theoretical studies by means of numerical simulations^9,10^. Observational strategies have been defined trying to detect new ETs but all the dedicated surveys performed so far have failed to discover any new member of this population^11–14^, including an in situ survey^15^ performed by the OSIRIS-REx spacecraft within the *L*_4_ region and observations^16^ of *L*_5_ by the Hayabusa2 spacecraft on its way to asteroid (162173) Ryugu. Despite failing in the detection of new ET asteroids, some of these surveys provided population constraints regarding their number and their size^12–15^.

The reason behind this low discovery success rate is related to the unfavorable viewing geometry of an object orbiting Earth-Sun’s *L*_4_ or *L*_5_ points as seen from our planet^17^. In short, these objects are often observable very close to the Sun (i.e., at low Solar elongations) and under large phase angles (the Sun-object-observer angle), meaning that a significant fraction of the object is shadowed as seen from the Earth, which in turn implies the object being faint. Under such geometries, observations must be acquired at high airmass, where seeing is typically worse, which, together with higher background from zodiacal light, further increases the difficulty of these searches. For both ETs known to date, opportunities for better observing geometries at larger solar elongations might exist thanks to their higher eccentricity values.

Asteroid 2020 XL_5_ was discovered by the Pan-STARRS1 survey on 2020 December 12. Shortly after the discovery, follow-up observations were gathered from different stations, allowing for an initial orbit determination. The orbit behavior suggested that 2020 XL_5_ could have been a candidate to become the second known ET, but the orbit uncertainty due to the short arc covered with observations was still too large to confirm a current Trojan engagement with Earth^18^.

Here we present the results of our study based on our new observations of 2020 XL_5_ which confirms that 2020 XL_5_ will be an ET for at least 4000 years.

## Results

### Orbit stability

The nominal orbit of 2020 XL_5_ has been computed at the epoch MJD = 58444.1 using the European Space Agency’s (ESA) AstOD orbit determination software^19,20^, based on the methods described in the literature^21^, taking as input the full observations dataset described in the observation sections of Methods. The resulting Keplerian orbital elements and their uncertainties are presented in Table 1. The dynamical model used in the orbit determination and propagation takes into account the *N*-body gravitational attraction of the Sun, eight planets, the Earth’s Moon, and the parameterized post-Newtonian relativistic contribution and the oblateness of Sun and Earth. The software makes use of the JPL Planetary and Lunar Ephemerides DE431^22^.

**Table 1:** Orbital elements.

Element	Value	1*σ* uncertainty	Unit
*a*	1.00070559767	5.61 × 10^−9^	au
*e*	0.387220870	1.56 × 10^−7^	
*i*	13.8458718	1.58 × 10^−5^	deg
Ω	153.6128174	5.03 × 10^−5^	deg
*ω*	87.9797957	4.25 × 10^−5^	deg
*M*	258.3840814	2.25 × 10^−5^	deg

Keplerian orbital elements of 2020 XL_5_ and their uncertainties, at the epoch MJD = 58444.1, computed with the European Space Agency’s (ESA) AstOD orbit determination software^19,20^, based on the methods described in the literature^21^, taking as input the full observations dataset described in the observation sections of Methods.

**Fig. 1:**
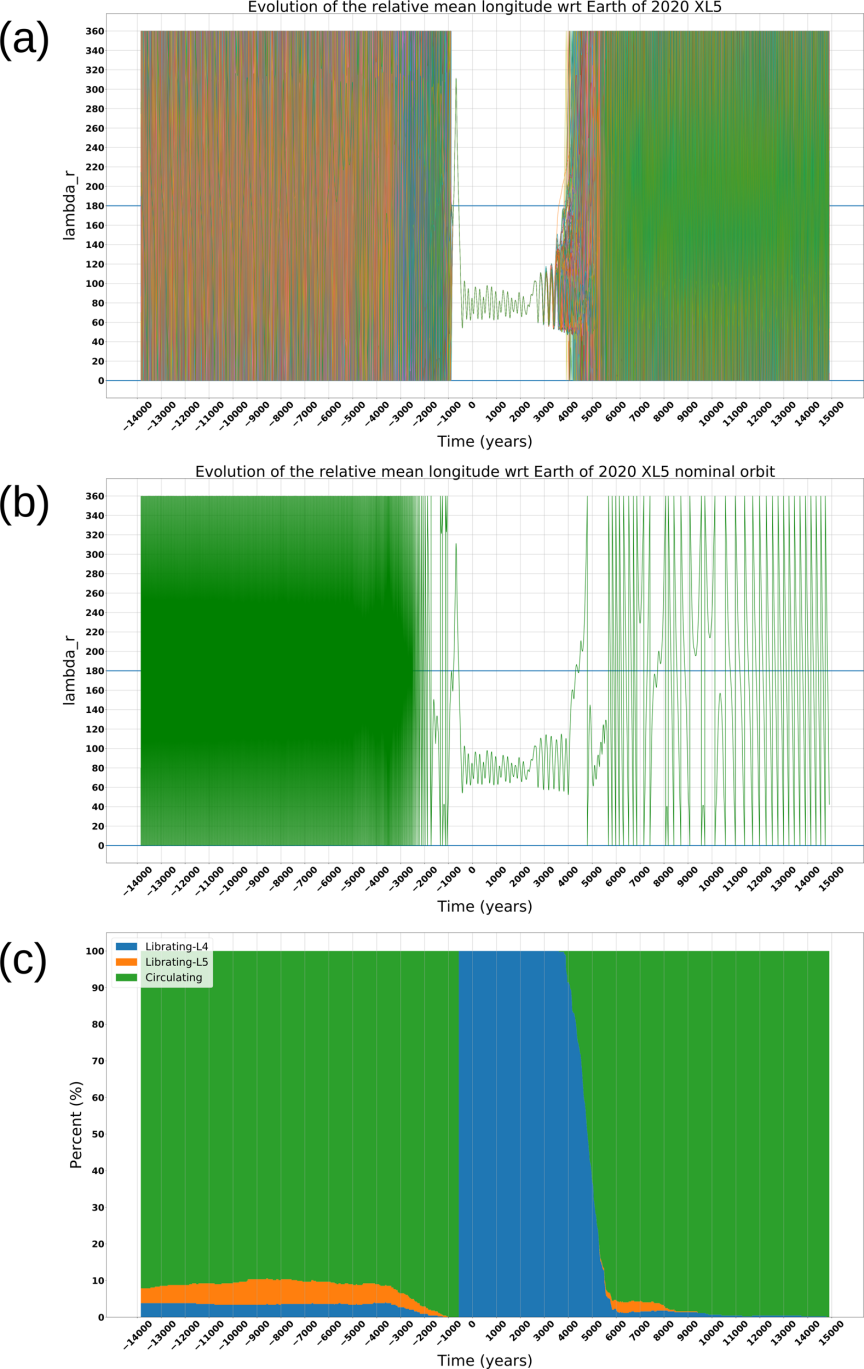
Mean longitude evolution analysis. **a** The relative mean longitude (*λ*_*r*_) evolution of the 2020 XL_5_ nominal orbit as in Table 1 and clones orbit over 29,000 years, where each clone orbit is represented by a different color, while the green line represents the nominal orbit. **b** Evolution of *λ*_*r*_ for the nominal orbit of 2020 XL_5_ over 29,000 years. **c** Stack plot representing the behavior of the nominal orbit and the 800 clone orbits. In the plot the time *t*_0_ = 0 is the mean epoch of the orbits. Source data are provided as a Source Data file.

In order to investigate the stability of the object in the *L*_4_ Lagrange point of the Earth, we performed a set of numerical simulations by integrating the nominal orbit, together with 800 clone orbits sampling its uncertainty, over a time span of ~29,000 years. The integration of the orbits has been performed using the ESA AstOD orbit determination software. The 800 clone orbits have been computed by sampling the orbit covariance matrix. The Trojan-like behavior of an object is seen in a reference frame co-rotating with the Earth’s orbital motion^23^. The key parameter to quantify the state is the relative mean longitude *λ*_*r*_ is defined as the difference between the mean longitude of the asteroid *λ*_*a*_ and the mean longitude of the Earth *λ*_*E*_: when the resonant angle *λ*_*r*_ librates around 60°, i.e., 0° < *λ*_*r*_ < 180° the asteroid is called an *L*_4_ Trojan, when the resonant angle *λ*_*r*_ librates around 300°, i.e., 180° < *λ*_*r*_ < 360° the asteroid is called an *L*_5_ Trojan, when *λ*_*r*_ circulates then the asteroid leaves the Trojan-like orbit^24^. Nevertheless, Trojans can have a displacement of a maximum of $\omega =2{5}^{\circ }11^{\prime}$ from the typical equilateral location for eccentric orbits^25^. We found that in the case of 2020 XL_5_ the mean longitude (*λ*_*r*_) evolution of the nominal orbit in Table 1 shows a transient ET behavior for this object (see Fig. 1). We define *t*_0_ = 0 as the mean epoch of the observations arc (corresponding to November 2018). The plot shows that before the time *t*_1_ ≃ −500 years, *λ*_*r*_ circulates, and therefore the asteroid could not be considered to be in a Trojan-like orbit. Starting from *t*_1_, 2020 XL_5_ is an *L*_4_ ET librating around *λ*_*r*_ ~ 75°, being stably located at the Lagrangian point for a time interval of about 4500 years, until the time *t*_2_ ≃ 4000.

**Fig. 2:**
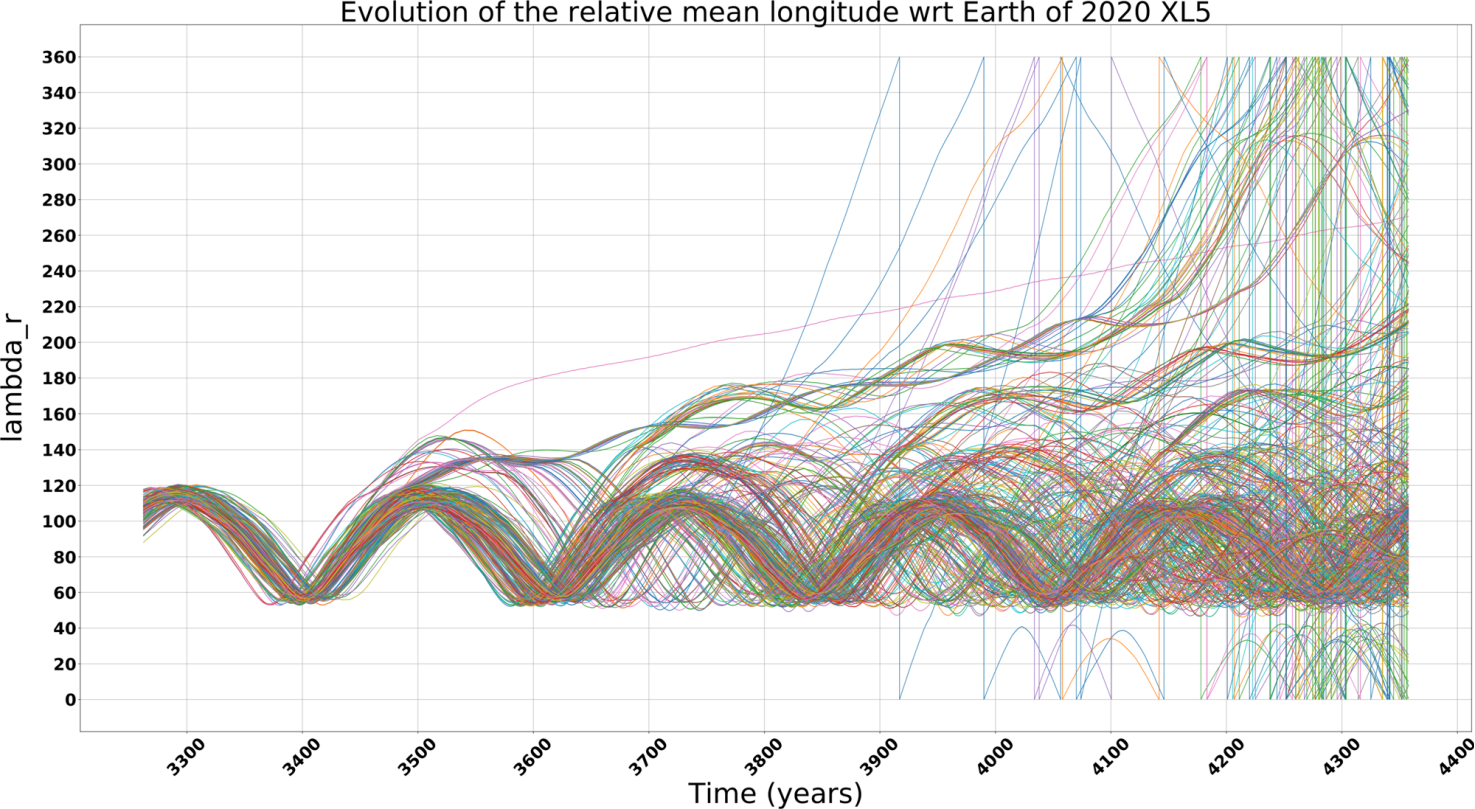
Beginning of the non-deterministic regime. The relative mean longitude (*λ*_*r*_) evolution of the 2020 XL_5_ nominal orbit as in Table 1 and clones orbit from year ~3300 until year ~4300, where each clone orbit is represented by a different color. Some clone orbits escape from the stability before the nominal one. Source data are provided as a Source Data file.

The observed stability time interval of the clone orbits appears to be consistent with that of the nominal orbit, as shown in Fig. 1, where a different color for each clone orbit has been used, with the green line representing the nominal orbit. Some clone orbits escape from stability before the nominal one, as shown in the zoomed Fig. 2.

To better visualize the results we generated a stack plot classifying the behavior of the nominal and clone orbits in three different states: librating around *L*_4_, librating around *L*_5_, and circulating (Fig. 1). The stack plot covers the entire time span of the simulations, with the time *t*_0_ = 0 set as the mean epoch of the observation arch. The plot shows that some orbits begin to show instability after time *t* ~ 3500, and by time *t* = 5000 less than 40% of the orbits are still in *L*_4_. Integrating backward, all the orbits become unstable by time *t* ~ −500, and less than 5% of orbits are located in *L*_4_ by *t* ~ −2000 and earlier. Moreover, the plot shows that less than 10% of the orbits are librating around *L*_5_ both before *t* ~ −1000 and during the interval between *t* ~ 6000 and *t* ~ 8000.

These transitions between libration points are a well-known behavior for objects in co-orbital motion, especially for those in orbits with large enough eccentricity and/or high enough inclination^26^.

### Physical characterization

**Fig. 3:**
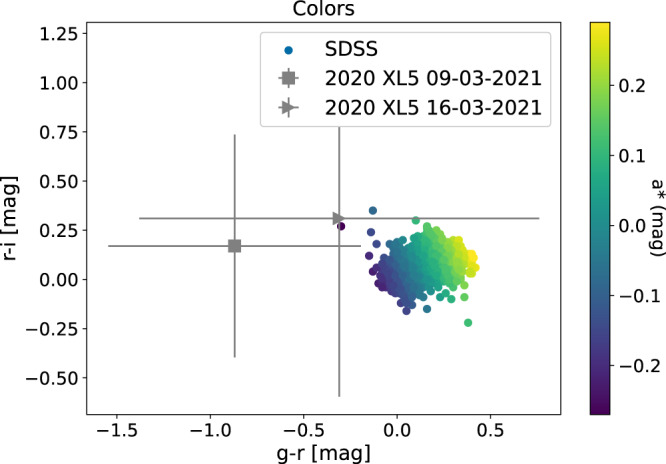
Color indices. The g-r and r-i colors indices of 2020 XL_5_ from the two observing nights, the color scale is according to the SDSS a* parameter. Gray lines represent one sigma color uncertainty. The 2043 color points represent 1308 asteroids from all taxonomic types present in the SDSS MOC DR3 catalog. The C-complex objects typically have a* < 0 with an average value of −0.1 and the S-complex object a* > 0 with an average value of 0.15^27^. 2020 XL_5_ is located in the C-complex area. Source data are provided as a Source Data file.

In addition to the orbital analysis discussed above, we also investigated the physical characteristics of 2020 XL_5_, by photometrically analyzing the new imaging data gathered during the 2020–2021 apparition and the precovery datasets. The SNR of the target was not sufficient to perform time-series photometry, since the object was only marginally detectable on single images, and could only be measured after stacking multiple frames. Moreover, since the observations were obtained close to dawn and with the telescope pointing very low over the horizon due to the object’s proximity to the Sun, the background signal increased very fast from frame to frame, and only the first 4 images for each band were useful for photometric purposes. Nevertheless, we obtained V-band photometry on 2021 February 22, Sloan g′, r′, and i′ photometry on 2021 March 9, and Sloan r′ photometry on 2021 March 13 and 16. Using these measurements, we investigated the taxonomy of the object; it is compatible with the C-complex, as shown in Fig. 3, although the low SNR of the measurements results in a large uncertainty for all the determined colors. The computed a* color is a* = −0.9 ± 0.6 for the first night and a* = −0.4 ± 1.0 for the second night, which are compatible with C-complex objects. The S-complex objects typically have a* > 0 and on average a* ~ 0.15 and the C-complex objects a* < 0 with average value of a* ~ −0.1^27^. In addition, deviations due to lightcurve amplitude effects could not be corrected, since the spin state of the object is still unknown. In light of the significant uncertainty of the photometric measurements, and the impossibility to compensate for rotational effects, this taxonomic classification is to be seen as provisional, and needs to be confirmed by further observations, ideally when the object presents a more favorable viewing geometry. We further investigated the photometric behavior of the object by analyzing the dependence of its brightness on the phase angle at the different nights of observations, to obtain the object’s phase curve (see Methods subsection Phase curve). After calibrating the data we obtained ${H}_{r}=18.5{8}_{-0.15}^{+0.16}$ for a C-type asteroid^28^ with ${G}_{{1}_{r}}^{* }=0.83$ and ${G}_{{2}_{r}}^{* }=0.02$. Thus, assuming a C-complex class, and therefore an albedo of 0.06 ± 0.03^29^, we estimate the diameter of 2020 XL_5_ as $1.1{8}_{-0.08}^{+0.08}\,{{\mbox{km}}}\,$. The uncertainty interval corresponds to the 16th and the 84th percentile, encompassing the 68% of the underling distribution of possible values. The inferred diameter for 2020 XL_5_ is larger than the value known for the first ET asteroid, 2010 TK_7_, which was estimated^8^ to have a diameter of ~0.3 km.

### Delta-v budget

ETs, among the Earth co-orbital objects, are considered to be potential candidates for rendezvous and even sample return missions, due to the low-energy requirements expected, as shown in previous works^30^ with a theoretical population of ETs. Therefore, we decided to investigate the required delta-v budget for both a rendezvous and a fly-by mission to the two ETs known, 2020 XL_5_ and 2010 TK_7_, in order to decide if they would be good candidates for a mission. The pykep tool^31^ used for the analysis is described in the Methods subsection Delta-v budget.

**Fig. 4:**
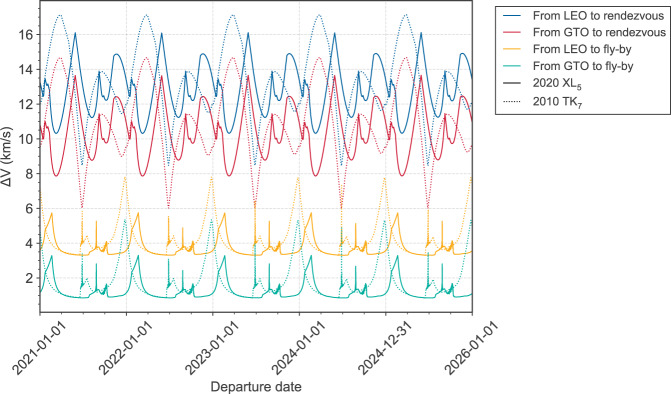
Delta-V of trajectories. Minimum delta-v trajectories to 2020 XL_5_ (solid line) and 2010 TK_7_ (dotted line) for the next 5 years. Four different scenarios are considered: departure from LEO and/or GTO orbit, and a rendezvous and/or fly-by mission to the asteroid. Source data are provided as a Source Data file.

Figure 4 shows, for both objects, the minimum total delta-v required for each departure date and each of the considered scenarios, i.e., a launch from Low Earth orbit (LEO) or Geostationary transfer orbit (GTO), and a space mission to a rendezvous or a fly-by with the asteroid.

First, for a rendezvous mission to 2020 XL_5_, the absolute minimum total delta-v is estimated to be between 7.9 and 10.3 km/s, depending on the launch conditions. Launching from LEO directly to escape is very expensive, and thus, the resulting required delta-v budget is not feasible. Getting to the GTO orbit via a shared launch significantly reduces the delta-v down to a value lower than 8 km/s, but it is still too high to be considered an ideal target for a rendezvous mission.

When comparing the results for 2020 XL_5_ to the other known ET, we can see that the latter presents slightly lower values for the absolute minimum total delta-v, which is estimated to be between 6 and 8.5 km/s, also far from the expected very low-energy requirements for rendezvous to theoretical ETs^30^. The main reason for such large velocities is the relatively high inclination of both 2020 XL_5_ and 2010 TK_7_, resulting in an additional plane-change maneuver that is extremely costly. In a previous large-scale statistical survey of delta-v to Earth co-orbital asteroids, similar conclusions were derived regarding the importance of inclination when considering a low delta-v budget to rendezvous asteroids with Earth-like orbits^30^. It might be preferable to consider another Earth co-orbital object closer to Earth’s orbital plane, or instead of a rendezvous mission, consider a fly-by mission to the asteroid. Therefore, we conclude that neither of the known ETs are good candidates for a space mission.

Figure 4 also shows the total delta-v required to perform a fly-by, which is significantly lower than for a rendezvous with the asteroid, since there is no need to match the asteroid’s orbit and hence no additional maneuver is performed. In addition, similar absolute minimum total delta-v values are obtained for both ETs, between 0.9 and 3.3 km/s depending on the launch conditions, making both of them potential fly-by targets. It is however important to highlight that 2020 XL_5_ presents a flatter minimum total delta-v with respect to the departure date. Therefore, 2020 XL_5_ might be a better candidate for a fly-by mission to an ET since it provides more flexibility to choose a suitable launch date.

## Discussion

Several observational surveys have been devoted to discover ETs near the L4 and L5 points^12–16^. Despite these efforts, only two objects have been discovered so far: 2010 TK_7_ and 2020 XL_5_. However, both asteroids are transient ETs, meaning that their stability around L4 has been shown to be in the scale of thousands of years, far from the stability time-scale of a theoretical primordial ET population, which are thought to be remnants from the Earth’s formation period^32^. Although no primordial ETs has been found yet, some constraints have been provided on their population. The most recent and restrictive values on their magnitude limit for L4^14^ are *N*_*ET*_ < 1 for *H* = 13.93, *N*_*ET*_ < 10 for *H* = 16, and *N*_*ET*_ < 938 for *H* = 22, while for L5^13^ are *N*_*ET*_ < 1 for *H* < 15.5, *N*_*ET*_ = 60 − 85 for *H* < 19.7, and *N*_*ET*_ = 97 for *H* = 20.4.

The discovery of a second ET asteroid may enhance our knowledge of the dynamics of this elusive population. By comparing the orbital nature of the two ETs known so far, we can better understand the mechanisms that allow for their transient stability. For instance, the librating point of both asteroids is displaced from the expected 60° due to their inclined and eccentric orbits. This might suggest that captured ET asteroids may likely be found in orbits displaced from the libration points.

Regarding the physical characterization of 2020 XL_5_, the improvement of its orbit and therefore its ephemeris to the arc-second level provided by this work is opening new interesting observational possibilities. In particular, it is now possible to plan observations with instruments having small fields of view. For instance, gathering new photometric data of the object during its yearly favorable observing window from November to December, will help to reduce the uncertainty on the color indices and therefore enhance its taxonomic classification. More specific studies resulting from infrared or spectroscopic observations will also enhance our knowledge of this dynamically exotic object. The latter will provide better constraints on its size estimation and composition. If follow-up observations confirm its C-complex nature, one reasonable explanation of this body’s orbit would be a transient capture from the main belt, after being ejected from the main belt by any of the 2:1 and 5:2 resonance complex with Jupiter^33^. Nonetheless, it has been shown^34^ that the Hungaria region could be a possible source of co-orbital bodies in the inner Solar System, with libration periods around ~10 kyr, in agreement with our results for 2020 XL_5_. On top of that, it has been found^35^ that the Hungaria region is mostly dominated by C and S-types, especially at small sizes, which is also consistent with our taxonomic classification of 2020 XL_5_.

Future surveys of the L4 and L5 regions will allow to derive tighter constraints on the ET populations and, maybe, discover their primordial bodies. Their study could help enhance the Solar System evolution models and therefore can be very valuable to understand its formation. Discovering ETs having lower orbital inclination and eccentricity might have another important implication: unlike 2020 XL_5_, objects librating near to the Lagrangian points with low inclinations could be reached from the Earth with a very low delta-v budget^30^. Therefore these objects may become ideal targets for space missions and, in the more distant future, to settle human bases or install scientific hardware that would benefit from their peculiar location.

## Methods

### New observations

The astrometric dataset of 2020 XL_5_, including information about the telescopes and the observing conditions, is presented in Table 2. In the following section, we describe the observations performed in our follow-up observation campaign for each of the telescopes used.

**Table 2:** Astrometry.

Date (UTC)	*α* (hh:mm:ss.sss)	*δ* (dd mm ss.ss)	*λ* (^∘^)	*β* (^∘^)	*l* (^∘^)	*b* (^∘^)	Δ*RA* (”)	Δ*Dec* (”)	*G*	MPC Code	Elong. (^∘^)	Phase (^∘^)	Airmass	Seeing (”)
2012-12-23.625791	12:58:16.435	−16 01 49.48	200	−9	305	47	0.112	0.100	21.32	F51	72.6	69.7	1.5	1.1
2014-11-04.369318	09:30:14.113	−00 38 50.31	145	−15	234	34	0.12	0.12	22.1	W84	77.0	49.6	1.4	1.2
2014-12-10.656600	11:42:30.865	−11 45 23.20	181	−13	277	48	0.122	0.129	22.09	F51	77.9	62.0	1.2	0.9
2014-12-11.657942	11:47:17.980	−12 05 29.58	182	−12	279	48	0.070	0.141	22.57	F51	77.6	62.5	1.2	1.0
2014-12-30.644316	13:36:45.039	−17 50 34.15	209	−7	318	44	0.485	0.100	20.97	F51	69.9	73.8	1.5	1.7
2014-12-30.647599	13:36:46.373	−17 50 37.07	209	−7	318	44	0.282	0.111	21.43	F51	69.9	73.8	1.5	1.7
2014-12-30.650895	13:36:47.664	−17 50 39.97	209	−7	318	44	0.321	0.194	21.08	F51	69.9	73.8	1.5	1.7
2014-12-30.654178	13:36:48.984	−17 50 42.77	209	−7	318	44	0.323	0.084	21.17	F51	69.9	73.8	1.5	1.7
2015-01-01.641762	13:50:24.755	−18 17 27.09	212	−6	322	42	0.156	0.127	21.37	F51	68.7	75.1	1.6	2.0
2015-01-01.646383	13:50:26.660	−18 17 30.71	212	−6	322	42	0.765	0.219	21.03	F51	68.7	75.1	1.5	2.0
2015-01-01.651037	13:50:28.563	−18 17 34.30	212	−6	322	42	0.249	0.163	21.38	F51	68.7	75.1	1.5	2.0
2015-01-01.655680	13:50:30.493	−18 17 37.92	212	−6	322	42	0.160	0.224	21.41	F51	68.7	75.1	1.5	2.0
2017-10-24.497090	08:57:38.428	+01 59 16.79	136	−15	227	29	1.0	1.0	22.3	G96	75.3	47.3	1.4	4.0
2019-10-27.508268	09:02:45.663	+01 22 05.46	138	−15	228	30	1.0	1.0	21.5	G96	76.3	47.8	1.3	2.6
2021-02-09.256526	18:25:55.70	−15 54 21.1	276	7	16	−2	0.8	0.8	—	J04	44.6	88.6	3.6	2.5
2021-02-09.267424	18:25:59.81	−15 54 11.4	276	7	16	−2	0.8	0.8	—	J04	44.6	88.6	3.1	2.5
2021-02-09.278810	18:26:04.09	−15 54 00.0	276	7	16	−2	0.8	0.8	—	J04	44.6	88.6	2.7	2.5
2021-02-22.533309	19:44:25.728	−12 28 24.00	296	9	28	−17	0.246	0.248	21.2	G37	38.9	79.6	4.2	2.9
2021-02-22.536635	19:44:26.820	−12 28 20.97	296	9	28	−17	0.235	0.229	21.2	G37	38.9	79.6	3.9	2.8
2021-03-09.397932	21:03:34.235	−08 47 40.59	316	8	40	−33	0.094	0.066	21.8	I33	33.8	63.8	3.6	0.9
2021-03-09.400223	21:03:34.940	−08 47 38.44	316	8	40	−33	0.087	0.079	21.8	I33	33.8	63.8	3.5	0.8
2021-03-09.403063	21:03:35.789	−08 47 35.95	316	8	40	−33	0.155	0.137	21.2	I33	33.8	63.8	3.3	1.4
2021-03-09.405788	21:03:36.626	−08 47 33.57	316	8	40	−33	0.102	0.072	21.5	I33	33.8	63.8	3.1	1.0
2021-03-14.408157	21:28:45.981	−07 33 20.58	322	7	45	−38	0.133	0.128	—	I33	32.3	58.3	3.3	1.6
2021-03-17.409321	21:43:29.790	−06 48 26.91	326	6	49	−41	0.5	0.5	—	I33	31.5	55.2	3.4	1.0

Astrometric measurements of 2020 XL_5_ from precovery archival data and follow-up at the discovery apparition. The table presents the astrometric measurements, their 1*σ* uncertainties in both coordinates, and the Gaia *G* magnitude for each measurement, followed by the MPC code of the station from which it has been obtained. The remaining columns present the observability circumstances at the time of each observation.

In order to extend the observed arc of 2020 XL_5_ into 2021, we gathered optical images of 2020 XL_5_ with two 4 m class telescopes (the Southern Astrophysical Research telescope and the Lowell Discovery Telescope) and a 1.0 m telescope (ESA’s Optical Ground Station) from 2021 February 9 to 2021 March 16, covering an additional orbital arc of 35 days.

The target’s viewing geometry and observational circumstances were extremely challenging during the time of all these observations: the target was very close to the Sun (low elongation), and also overlapping with the galactic plane earlier in the observation period.

We designed our observing strategy for the purely astrometric datasets prioritizing the goal of achieving the minimum signal-to-noise ratio (SNR) necessary in order to make the object measurable from an astrometric point of view. We, therefore, used broad-band filters in all our observations and pointed to the object as soon as it reached the minimum elevation allowed by the telescopes’ specifications. All in all, we had a few minutes per night to observe the object until the background quickly started saturating due to twilight.

Additionally, the length of the exposure times was limited by the apparent motion of 2020 XL_5_. Therefore to achieve the best possible astrometric accuracy, while still keeping the ability to track non-linearly on the motion of the object, and reject bright overlapping stars, we decided to limit our single exposures to at most 20s. Astrometric measurements were then extracted from stacked images for each night, whereas a quality test for the background levels was performed to select the frames qualifying for the photometric analysis.

#### Optical ground station (OGS)

ESA’s Optical Ground Station (OGS) 1.0 m telescope (MPC code J04) in Tenerife, Canary Island, Spain, is regularly used by ESA’s Planetary Defence Office to obtain astrometric observations of NEOs. In the context of such routine monitoring and follow-up activities, our team obtained and reported observations of the target on 2020 December 13, providing observational confirmation of the object’s existence just 14 h after the initial discovery. Other stations reported additional data over the following nights, but the observational coverage available at the Minor Planet Center ended in early 2021 January. By the end of the month, the object’s trajectory brought it towards the skyplane location of the galactic center, and observations became consequently more challenging. We nevertheless decided to attempt further observations in early February with the OGS telescope. The field containing the object was exposed on three consecutive mornings, from 2021 February 9 to 11. On the first night, we could detect a possible faint candidate on a small subset of frames not significantly affected by background stars. On the subsequent nights, the stellar confusion of the field was too significant to achieve any detection. All the OGS observations were obtained with the ESA Space Debris Camera 2 (SDC2), equipped with a 4k imager used in 2 × 2 binning mode and sidereal tracking. This instrument configuration results in a $47.5^{\prime}$ FoV, with 1.39*″* binned pixels, optimally sampling the ~2.5*″* to 3*″* FWHM of the system’s PSF for astrometric purposes.

#### Lowell discovery telescope (LDT)

**Fig. 5:**
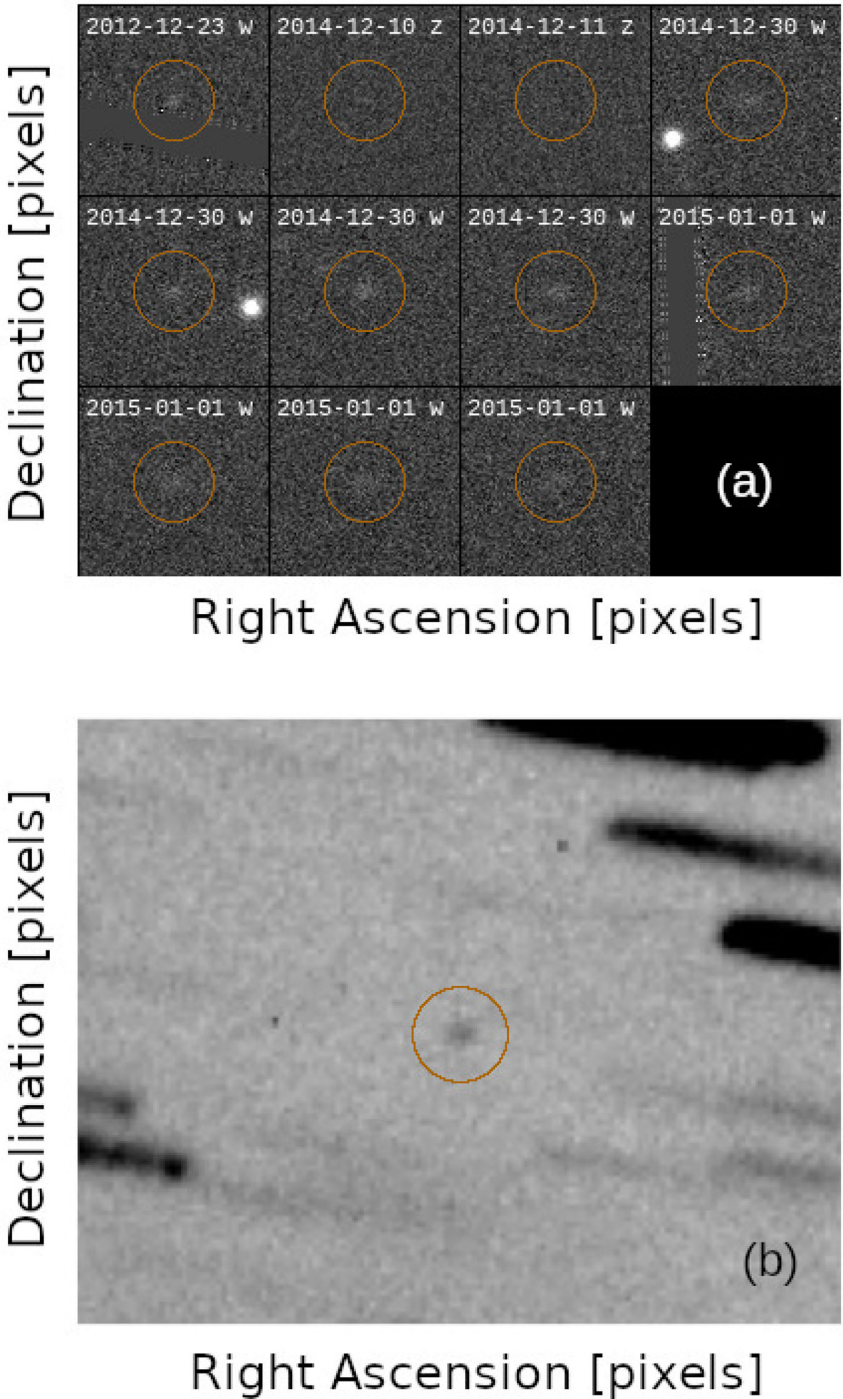
Example of detections. **a** A mosaic showing the Pan-STARRS pre-discovery observations of 2020 XL_5_. The orange circles highlight the position of the object. **b** Overall stack of the 13 frames obtained with the Lowell Discovery Telescope on 2021 February 22. The orange circle highlights the position of the object.

Observations at the 4.3 m Lowell Discovery Telescope (G37) were obtained on 2021 February 22 during astronomical twilight when 2020 XL_5_ was at an airmass between 4.2 and 3.8. We used the Large Monolithic Imager (LMI)^36,37^. LMI is a e2v CCD231-SN-10382-14-0 of 6144 × 6160 15 μm pixels. On the LDT, LMI provides a field of view of $12.3^{\prime} \times 12.3^{\prime}$ with a pixel scale of 0.12*″*/pixel when operated unbinned. For the 2020 XL_5_ observation we used a binning mode of 5 × 5 providing a pixel scale of 0.60*″*/pixel. We obtained 13 individual exposures of 20 s each. The telescope was tracking at a non-sidereal rate matching the motion of the asteroid of 3.44*″*/pixel providing star trails of 1.15*″* that are much smaller than the ~3*″* seeing at such high airmass. The observations were performed in the VR filter (bandpass from 0.480 ± 0.005 to 0.721 ± 0.005 μm). The resulting stacked image is shown in Fig. 5.

#### Southern astrophysical research (SOAR)

We used the 4.1 m Southern Astrophysical Research Telescope (I33) on the nights of 2021 March 9 (NOIRLab program 2018B-927, P.I., S. Zepf, Michigan State, University), 13 (NOIRLab Astronomical Event Observatory Network (AEON) 2021A queue), and 16 (Brazil DDT night, PI: L. Fraga). We used the Goodman optical imager which provides a field of view of $7.2^{\prime}$ and a pixel scale of 0.15*″*/pix. Observations were very challenging due to the extremely low solar elongation of the object at that time (between 32° and 34° away from the Sun). As a result, the object was observable only during a few minutes before dawn and very close to the horizon (15° elevation), with an airmass between 3.7 and 3.3. We used the Goodman HTS imaging camera equipped with SDSS filters and 3 × 3 binning mode in order to reduce the readout time. We tracked non-sidereally and used short exposures (20 s) for each of the g′, r′, and i′ filters. The background was quickly saturating and only the first four frames of each band were useful for photometric analyses.

#### Additional observational attempts

We also attempted observations of 2020 XL_5_ using the 0.8 m Joan Oró (TJO) telescope (MPC code C65) and the 2.2 m Calar Alto (CAHA) telescope (MPC code 493) on 2021 February 11 and 2021 March 2 respectively. However, on both attempts, we could not confidently detect the object, even after stacking all the frames, and therefore we are not including these data in the analyses presented in this work.

### Precovery observations

The new observational data gathered by our team and discussed above, combined with the astrometry available at the Minor Planet Center, cover the entire observable arc from the time of the earliest submitted precovery observation (2020 November 26, by code G96), to the disappearance of the object into solar conjunction (elongation <30°, at the end of 2021 March), which marked the end of the discovery apparition. Overall, the arc covers almost 111 days, sufficient for a rough analysis of the stability of the object, but not ideal for a long-term study of the behavior of the object^18^.

In order to increase the observed arc without the need to wait for an additional future apparition, we performed a thorough search for precovery detections in archival data. We began with the archive of the Catalina Sky Survey: the search revealed a promising field exposed with the 1.5 m Mt. Lemmon telescope (G96) on 2019 October 27, at a time when the object had an ephemeris uncertainty of just ±5*″* (1*σ* confidence) based on the data from the 2020–2021 apparition. A careful analysis of the area of the images corresponding to the prediction revealed a possible faint candidate with a signal-to-noise ratio (SNR) of ~3.

Despite not being a convincing detection, we temporarily assumed its correctness and used it to extend our search further back in time. Another promising field was located, exposed by the same telescope on 2017 October 24. The uncertainty of the ephemeris at that time, assuming the correctness of the 2019 detection, was only ±2.3*″*. No source was visible in the single images, but another very faint source with SNR ~3 appeared in the stack of all four frames of that night. These two tentative detections, individually not sufficiently strong to claim a certain identification, when taken together provide reasonable evidence that they could indeed be real. Their orbit improvement potential was so great that they now made it possible to determine the object’s ephemeris to better than ±5*″* as far back as 2012, allowing for many additional precovery opportunities at multiple apparitions.

We, therefore, extended our search to two additional archives: the online repository of all images obtained by the wide-field DECam instrument with the 4.1m Víctor M. Blanco Telescope on Cerro Tololo, Chile, and the archive of the 1.8m Pan-STARRS survey. The DECam archive provided a solid detection compatible with the tentative G96 precoveries on 2014 November 4, while Pan-STARRS thoroughly confirmed the chain of precovery observations providing eleven additional measurements covering a time span from 2012 to 2015 (see Fig. 5).

### Orbit determination

Clone orbits used in the numerical simulations were generated applying the Cholesky method for multivariate normal distributions^38^. The clone orbits are generated using Python 3.6^39^, starting from the nominal orbit and its covariance matrix computed with the AstOD software. Alternatively, freely available software such as OrbFit^40^ can be used for the analysis.

The Cholesky method consists of the factorization of a Hermitian, positive-definite matrix, as the product of a lower triangular matrix and its transpose conjugate. In our case, the covariance matrix *C* is thus decomposed as *C* = *LL*^*T*^, where *L* is a lower triangular matrix. The Keplerian orbital elements **e**_**i**_ of the *i*-th clone orbit are then defined as follow:

1 $${{{{{{{{\bf{e}}}}}}}}}_{i}={{{{{{{{\bf{e}}}}}}}}}_{{{{{{{{\bf{0}}}}}}}}}+L{{{{{{{{\bf{r}}}}}}}}}_{{{{{{{{\bf{i}}}}}}}}},$$

where **e**_**0**_ is the vector of the orbital elements of the nominal orbit, and **r**_**i**_ is a 6-*dim* random vector, whose components are generated following a normal distribution with mean 0 and variance 1 (${{{{{{{\mathcal{N}}}}}}}}(0,1)$).

Since the uncertainties in the nominal orbital elements are very small differences between the initial conditions of the clone orbits and those of the nominal orbit are small as well.

Starting from these initial conditions, we have integrated the 800 clone orbits along a time span of ~29,000 years. Considering as initial time *t*_0_ = 0 the mean epoch of the observations, the forward propagation has been executed along 15,000 years, where this limit in time is defined by the JPL Planetary and Lunar Ephemerides DE431^22^ whilst the backward propagation has been executed for 14,000 years. As a second step, we studied the evolution of the relative mean longitude *λ*_*r*_ of 2020 XL_5_ with respect to the Earth. The relative mean longitude *λ*_*r*_ is defined as the difference between the mean longitude of the asteroid *λ*_*a*_ and the mean longitude of the Earth *λ*_*E*_: when the resonant angle *λ*_*r*_ librates around 60°, i.e., 0° < *λ*_*r*_ < 180° the asteroid is called an *L*_4_ Trojan, when the resonant angle *λ*_*r*_ librates around 300°, i.e., 180° < *λ*_*r*_ < 360° the asteroid is called an *L*_5_ Trojan, when *λ*_*r*_ circulates then the asteroid leaves the Trojan-like orbit^24^.

We have reproduced the same calculations using the public software OrbFit^40^, obtaining consistent results for the deterministic part of the orbital evolution.

### Validation of the orbital study method

**Fig. 6:**
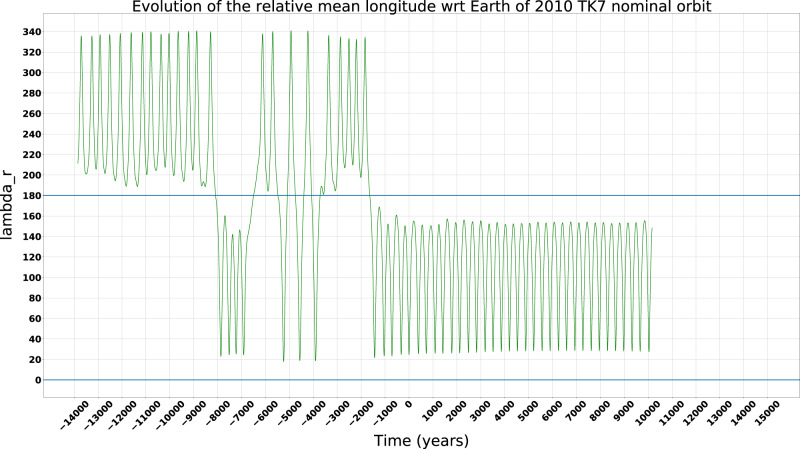
Mean longitude evolution analysis of 2010 TK_7_. Evolution of *λ*_*r*_ for the nominal orbit of 2010 TK_7_ along 29,000 years. Source data are provided as a Source Data file.

In order to validate the methods adopted during the analysis of the orbital stability of 2020 XL_5_, we applied the same approach also to 2010 TK_7_, the other known ET. In Fig. 6 we report the evolution of its *λ* over the integration interval. In this case, the *t*_0_ = 0 is set as the mean epoch of the observations arc of 2010 TK_7_, which corresponds to 2012 August. The results obtained on the stability in *L*_4_ are fully consistent with the ones reported in the literature^8,24^, confirming the validity of our computational methods and approach.

### Photometry

In order to perform our photometric measurements, we optimized the object’s SNR by creating different stacked images on the motion direction of 2020 XL_5_. In order to create the stacked images and to make the photometric measurements, we used the publicly available Tycho Tracker software^41^. For each night, we started selecting all the frames gathered for each filter to create a stacked image and measure the object’s SNR. We then repeated the process after removing the last frame ordered chronologically and we iterated the process until we were left with only four images to stack. We then compared the SNR values obtained for the different stacks and selected the image with the target having the highest SNR. With this process, we managed to obtain the best possible photometry, despite the nearly saturated background due to the twilight proximity. We then used 3.5 pixel apertures to measure the flux of 2020 XL_5_. As comparison stars, we selected all the solar analog stars in the field and we used the values of the ATLAS catalog^42^ to compute the absolute photometry for the different bands used in our observations.

In order to select the stacked images suitable for photometry, we applied a threshold filter of SNR >5. From the follow-up observations gathered with SOAR, we could obtain three $r^{\prime}$ measurements and two with $i^{\prime}$ and $g^{\prime}$ filters. Observations with LDT allowed us to extract a measurement in the *V* filter. We also checked the recovery data and managed to extract two $r^{\prime}$ measurements from the Pan-STARRS data. Unfortunately, DECam’s data had an SNR <3, which was not enough to extract any photometric measurement. The photometric measurements for the different bands and nights are presented in Table 3.

**Table 3:** Photometry.

*Date*	*α* (^∘^)	$r^{\prime}$	$i^{\prime}$	$g^{\prime}$	*V*	MPC Code
2014 December 30	74	21.41 ± 0.16				F51
2015 January 1	75	21.48 ± 0.16				F51
2021 February 22	80				21.76 ± 0.15	G37
2021 March 9	64	21.26 ± 0.42	20.99 ± 0.34	20.84 ± 0.42		I33
2021 March 14	58	20.85 ± 0.31				I33
2021 March 16	56	20.63 ± 0.43	20.22 ± 0.50	20.77 ± 0.98		I33

Photometric measurements of 2020 XL_5_ as defined in the text. The table presents the phase angle at the moment of observation and the measured photometry in r′, i′, g′, and V bands, together with the MPC code of the telescope used for the observation.

### Color indices

We estimated the reflectance values at the wavelengths of standard Sloan Digital Sky Survey (SDSS) filters following a procedure from the literature^43^. We first computed the solar-corrected^44^ colors and albedos, normalized at the r band, and their corresponding uncertainties.

Furthermore, we computed the a* parameter^45^ a* = 0.9285(*g* − *r*) + 0.3712(*r* − *i*) − 0.66204, which turns out to be a* = −0.9 ± 0.6 for 2020 XL_5_ on the first observing night, and is more consistent with C-complex objects^44^; on the other hand, we obtained a* = −0.4 ± 1.0 for the second night, compatible with all possible complexes^44^. Additionally, we plotted the *g* − *r* and *r* − *i* colors indices of 2020 XL_5_ together with objects from the SDSS MOV catalog release 3 (see Fig. 3). From the SDSS catalogs, we selected only those objects which fulfill the quality criteria as found in the literature^46^. The estimated colors lie outside the space occupied by most other asteroids from the SSDS but are offset towards the C-complex objects. The uncertainties of colors estimated from the second night are compatible with all possible taxonomic complexes.

### Phase curve

**Fig. 7:**
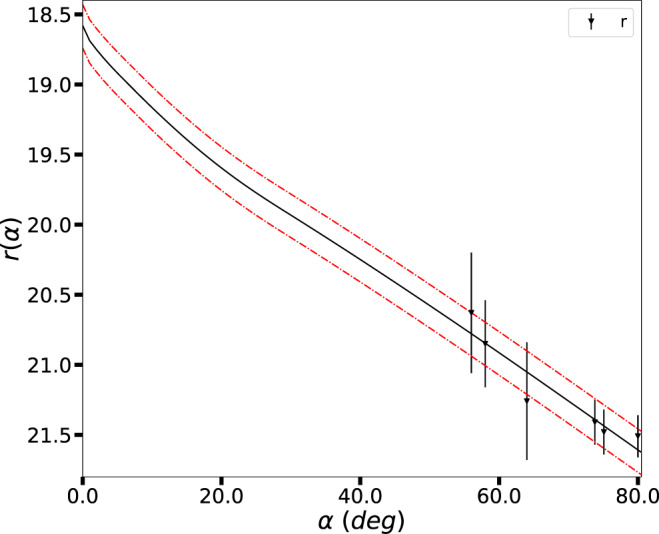
Photometric phase curve. Phase curve in the *r* filter of 2020 XL_5_ using the six observing nights. The dashed line indicates the adopted solution, while the dot-dashed line indicates the uncertainty interval of the absolute magnitude. Source data are provided as a Source Data file.

We used the magnitudes in the *r* filter to construct a phase curve for 2020 XL_5_ (see Fig. 7). We first normalized the magnitudes to unit distances to the Earth and the Sun according to $r(\alpha )=r(R,{{\Delta }},\alpha )-5{{{{{{\mathrm{log}}}}}}}\,(R{{\Delta }})$, where *α* is the phase angle, *R* is the heliocentric distance, and Δ the geocentric distance (both in au). We then applied the HG12* model^47^ using the online version of the tool^48^. Because of the large phase angles and the low coverage of the phase curve, the best fit we obtained assumes a single free parameter, the absolute magnitude, while *G*12* is fixed, according to different taxonomical types. In our case, the best fit was obtained for the P and C types. The error in the magnitudes is estimated using Monte Carlo methods and the lower (upper) uncertainty corresponds to the 16th (84th) percentile. Note that the last point in the phase curve, at about *α* = 80°, was obtained from the measured *V* magnitude using the transformations from Sloan’s ugriz to UBVRI^49^.

### Delta-v budget: patched-conics approach

Despite the proximity of the ETs orbits, they are still deep-space targets, and thus, any practical mission to this kind of orbits is likely to make use of gravity-assists and/or low-thrust solar electric propulsion (SEP). However, for the purpose of this paper, we limited our search to sub-optimal delta-v trajectories using a patched-conics approach with ballistic trajectory.

For the heliocentric segment of the transfer trajectory, we solved Lambert’s boundary value problem (BVP) in order to determine the Keplerian orbit that connects the spacecraft and the target asteroid in space in a given elapsed time. Lambert’s BVP solver is very computationally fast, which allows us to do a quick survey of ballistic transfers from Earth to 2020 XL_5_ for different departure dates and time-of-flights (TOF). For this paper, we used the multi-revolutions Lambert’s problem solver^50^ implemented within the pykep software^31^ by the mission analysis team at ESA/ESOC.

For the geocentric portion of the trajectory, the escape orbit is calculated starting from both a low Earth orbit (LEO) at approximately 300 km, and a geostationary transfer orbit (GTO) with a perigee altitude of also 300 km and the apogee at GEO altitude. In general, similar research works only deal with a simple launch from LEO; however, it is very expensive to go from LEO directly to escape with only chemical propulsion. For this reason, we also considered the GTO orbit, which is more common in actual space missions (especially if we assume a shared launch) and reduces significantly the cost of getting out of the Earth’s sphere of influence (SOI).

### Estimation of the total delta-v

The total delta-v for this mission is estimated as the sum of the delta-v required to go from launch conditions to Earth’s escape orbit, *dv*_1_, and the delta-v required to do the asteroid-rendezvous, *dv*_2_, which is zero for a fly-by mission. For a given transfer trajectory by Lambert’s problem, we can compute *dv*_1_ as the excess velocity required at the perigee of the starting orbit in order to enter an Earth’s escape trajectory with a velocity at infinity (*v*_inf_) equal to the relative velocity difference between the Earth and the calculated starting velocity of the transfer trajectory at the launch date. Similarly, *dv*_2_ can be computed as the velocity difference between the target asteroid and the calculated ending velocity of the transfer trajectory at the arrival date.

For each combination of departure date and TOF, we can compute a single set of *dv*_1_ and *dv*_2_. Therefore, in order to find the optimal transfer trajectory, we have performed a scan in TOF, between 180 days and 3 years and a time step of 1 day, and we selected the minimum total delta-v (*dv*_1_ + *dv*_2_) as the optimal solution for the departure date considered. We have repeated this process for the next departure date, which is about 0.5 days later. The launch window considered goes from 2021 January 1 until 2026 January 1. This time window is enough for the purposes of this study, since the results are completely periodic, and thus, similar results can be expected for other departure dates. Additionally, we have validated our results using the JPL Small-Body Mission-Design online Tool^51^, which provided very similar results.

## Data Availability

The datasets generated during and/or analysed during the current study are available from the corresponding author on reasonable request. DECam data are available in the NOIRLab Archive Astro Data Archive (https://astroarchive.noirlab.edu). Pan-STARRS data are available upon request to R. Wainscoat (rjw@hawaii.edu). Catalina Sky Survey data are available upon request to E. Christensen (eric@lpl.arizona.edu). Lowell Discovery Telescope data and SOAR data are available upon request to the corresponding author. Astrometric measurements are available on the MPC site (http://www.minorplanetcenter.net/db_search/show_object?object_id=2020+XL5). Source data are provided with this paper.

## Source data

Source Data

## Code availability

The ESA NEO Coordination Centre’s orbit determination and impact monitoring AstOD software used for the study of the orbit stability is proprietary (https://neo.ssa.esa.int/about-neocc). The Tycho Tracker software was used for data processing and photometric analysis (http://www.tycho-tracker.com). The pykep scientific library was used for the delta-v budget calculations (http://esa.github.io/pykep).
